# Genetic diversity of *DGAT1* gene linked to milk production in cattle populations of Ethiopia

**DOI:** 10.1186/s12863-022-01080-8

**Published:** 2022-08-10

**Authors:** Behailu Samuel, Dejenie Mengistie, Ermias Assefa, Mingue Kang, Chankyu Park, Hailu Dadi, Hunduma Dinka

**Affiliations:** 1 Department of Applied Biology, Adama Science and Technology University, P. O. Box 1888, Adama, Ethiopia; 2 Bio and Emerging Technology Institute, P. O. Box 5954, Addis Ababa, Ethiopia; 3 Department of Stem Cell and Regenerative Biotechnology, Konkuk University, Seoul, South Korea

**Keywords:** *DGAT1*, Ethiopian Cattle breeds, Genetic diversity, Evolution, Sequencing

## Abstract

**Background:**

Diacylglycerol acyl-CoA acyltransferase 1 (*DGAT1*) has become a promising candidate gene for milk production traits because of its important role as a key enzyme in catalyzing the final step of triglyceride synthesis. Thus use of bovine *DGAT1* gene as milk production markers in cattle is well established. However, there is no report on polymorphism of the *DGAT1* gene in Ethiopian cattle breeds. The present study is the first comprehensive report on diversity, evolution, neutrality evaluation and genetic differentiation of *DGAT1* gene in Ethiopian cattle population. The aim of this study was to characterize the genetic variability of exon 8 region of *DGAT1* gene in Ethiopian cattle breeds.

**Results:**

Analysis of the level of genetic variability at the population and sequence levels with genetic distance in the breeds considered revealed that studied breeds had 11, 0.615 and 0.010 haplotypes, haplotype diversity and nucleotide diversity respectively. Boran**-**Holstein showed low minor allele frequency and heterozygosity, while Horro showed low nucleotide and haplotype diversities. The studied cattle *DGAT1* genes were under purifying selection. The neutrality test statistics in most populations were negative and statistically non-significant (*p* > 0.10) and consistent with a populations in genetic equilibrium or in expansion. Analysis for heterozygosity, polymorphic information content and inbreeding coefficient revealed sufficient genetic variation in DGAT1 gene. The pairwise *F*_*ST*_ values indicated significant differentiation among all the breeds (*F*_*ST*_ = 0.13; *p* ≤ 0.05), besides the rooting from the evolutionary or domestication history of the cattle inferred from the phylogenetic tree based on the neighbourhood joining method. There was four separated cluster among the studied cattle breeds, and they shared a common node from the constructed tree.

**Conclusion:**

The cattle populations studied were polymorphic for *DGAT1* locus. The *DGAT1* gene locus is extremely crucial and may provide baseline information for in-depth understanding, exploitation of milk gene variation and could be used as a marker in selection programmes to enhance the production potential and to accelerate the rate of genetic gain in Ethiopian cattle populations exposed to different agro ecology condition.

## Background

The candidate gene, Diacylglycerol acyl-CoA acyltransferase (*DGAT*) activity was first described by Weiss and Kennedy [1] and *DGAT1* enzyme was found to play fundamental role in the metabolism of cellular triacylglycerol during physiological processes, such as intestinal fat absorption, lipoprotein assembly, fat tissue formation and lactation [2]. Functionally, the *DGAT1* gene was identified as one of at least two genes that encodes *DGAT* enzyme which catalyzes the final step of triglyceride synthesis in eukaryotic cells [3]. It became a functional candidate gene for lactation traits after studies indicated that mice lacking both copies of *DGAT1* are completely devoid of milk secretion, presumably due to deficient triglyceride synthesis in the mammary gland [4]. The fluorescence in situ hybridization and radiation hybridization method identified *DGAT1* having profound effect on milk production [5] and primarily responsible for milk fat variation in dairy animals [6]. Bovine *DGAT1* was the first identified gene of about 14,117 bp and 17 exons that encodes a protein with *DGAT* activity [2]. In the centromeric region of the bovine chromosome 14 [7] a missense mutation K232A (Lys232 → Ala) was shown to be significantly associated with variation in milk fat on exon 8 region [5].

According to the studies undertaken so far the reported haplotype number, haplotype diversity and nucleotide diversity for *Bos indicus* cattle were 2, 0.536 and 0.003 respectively [8] and gene diversity for *Bos indicus* cattle range from 0.02–0.50 for *DGAT1* gene [9]. Negative estimates of *F*_*IS*_ (inbreeding coefficient) was observed in Creole and Borgou cattle of Uruguay and Benin cattle respectively [10, 11]. Pairwise *F*_*ST*_ values for pooled subpopulations showed least divergence for *Bos indicus* breeds with high milk fat percentage for *DGAT1* gene [9]. Among the seventeen exons of *DGAT1* gene, exon 8 has previously been reported to be the most polymorphic and potentially affect milk composition and yield traits [5, 7, 12]. Moreover, studies reported that the *DGAT1* gene has been associated with regulation of the synthesis of Vitamin A and somatic cell count in lactating cattle [13, 14]. These all mentioned reports indicate *DGAT1* can be used as practical genetic markers for selective breeding of dairy cattle.

The effect of any identified polymorphism may differ across different populations or breed because of specific genetic backgrounds. Ethiopia has the largest cattle population in Africa and the fifth largest in the world and estimated to be about 70 million and indigenous cattle hold great promise and potential for milk production and constitute about 97.4% of the total cattle population [15]. Some census has witnessed an increase of 1.97% in population of milking cattle from 19.7 million to 22.5 million. For instance, Boran and Begait are *Bos indicus* zebu cattle breeds with a well-developed udder, long legs, and large humps, long teats and known for their milk, resistance to heat stress and tick infestation [16–18]. Moreover Fogera and Horro are *Bos indicus* Zenga (Zebu x Sanga) cattle breeds mainly characterized by their calm disposition and variable milk production [17–19]. To increase milk volume Boran cattle are crossed with Holstein–Friesian dairy breeds and Boran**-**Holstein crossbreds have increased lactation lengths, shorter calving intervals and calve at a younger age than the indigenous stock [20, 21]. Before the aforementioned marker is used for the genetic improvement of Ethiopian cattle productivity, its polymorphism should be clearly studied. Unfortunately, no study has been undertaken to understand the genetic diversity within a population, analysis of evolution, neutrality test and *DGAT1* gene population differentiation among Ethiopian cattle breeds that are critically important for future breeding programs. Therefore, the aim of this study was to characterize the genetic variability of exon 8 region of *DGAT1* gene in Ethiopian cattle breeds through sequencing.

## Results

### *DGAT1* gene genetic diversity in Ethiopian cattle breeds

**Fig. 1:**
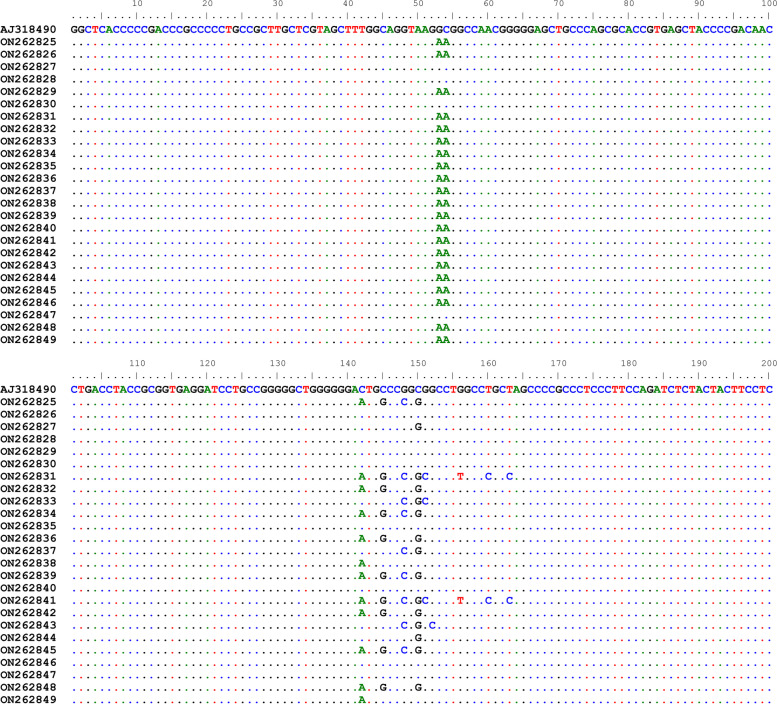
The nucleotide sequences alignment of studied breeds with the reference sequence from the NCBI data base (Ac.No.AJ318490). Boran**-**Holstein (ON262825**-**ON262828), Boran (ON262829**-**ON262833), Begait (ON262834**-**ON262838), Fogera (ON262839**-** ON262844) and Horro (ON262845**-**ON262849)

The nucleotide sequences of studied breeds were compared with the reference sequence from the GenBank Accession No.AJ318490 and variability of the region was visualized (Fig. 1).

The number of polymorphic sites (*S*) which is the measure of usable loci that show more than one allele per locus was analyzed. Accordingly, the value of *S* was higher in Boran (10) and lower in Begait (4). Horro and Boran**-**Holstein cattle had the same number of polymorphic sites (6). The nucleotide diversities (*π*) were relatively high in Begait breeds (0.010) whereas in the Horro breed a relatively low number of **π** (0.005) was observed. For all breeds, non-synonymous substitutions were less as compared to the synonymous substitutions (Table1).

**Table 1:** DNA polymorphism and evolution of *DGAT1* gene for considered breeds

Breeds	π	S	H	Hd	Ka	Ks	Ka/Ks
BR	0.009	10	5	0.507	0	0.014	0
BG	0.010	4	5	0.700	0	0.013	0
FG	0.009	9	6	0.514	0.0047	0.0062	0.758
HR	0.005	6	5	0.450	0	0.005	0
BHC	0.008	6	4	0.636	0.0037	0.01	0.37

Nucleotide diversity (π), number of polymorphic sites (S), haplotype number (H), haplotype diversity (Hd), synonymous (Ks), non-synonymous (Ka), Boran-Holstein (BHC), Boran (BR), Begait (BG), Fogera (FG ), Horro (HR)

Haplotypes were constructed for each breed and a total of 11 haplotypes were obtained. The haplotype values ranged from 6 (Fogera) to 4 (Boran**-**Holstein). Boran, Begait and Horro cattle had the same number of haplotype number (5).

The haplotype diversity (Hd) of the Begait and Boran**-**Holstein cattle were relatively higher as compared to the Horro. The haplotype diversity for Horro was 0.450 which was the lowest as compared to the rest of the breed (Table 1).

### Evolution of *DGAT1* gene among Ethiopian cattle breeds

The ratio between non-synonymous (Ka) and synonymous mutations (Ks) was calculated to have an insight on the evolution of *DGAT1* (Table1). The ratio of non-synonymous substitution (Ka) to synonymous substitution (Ks) was highest in Fogera with an estimated value of 0.758 and lowest in Boran, Begait and Horro with an estimated value of 0.00.

### *DGAT1* gene genetic parameters and neutrality tests in Ethiopian cattle breeds

For analysis of genetic diversity within a population, minor allele frequency (MAF), polymorphic information content (PIC), average expected (*H*_*e*_) and observed heterozygosity (*H*_*o*_) values, HWE in terms of *F*_*IS*_ coefficient and neutrality tests were estimated. The observed heterozygosity (*H*_*o*_) ranged between 0.157 in Boran**-**Holstein to 0.413 in Begait cattle (Table 2).

**Table 2:** Genetic parameters estimates and neutrality tests for the considered breeds

Breeds	***N***	**MAF**	***H***_***o***_	***H***_***e***_	**PIC**	***F***_***IS***_	**Fu’s *Fs***	**Tajima’s *D***
BR	17	0.129	0.259	0.176	0.148	-0.446	0.941^ ns^	-1.204^ ns^
BG	16	0.206	0.412	0.256	0.200	-0.591	0.461^*^	2.364^*^
FG	17	0.153	0.306	0.196	0.163	-0.538	-0.631^ ns^	-0.956^ ns^
HR	16	0.119	0.238	0.146	0.118	-0.606	-1.037^ ns^	-1.214^ ns^
BHC	23	0.078	0.157	0.120	0.101	-0.283	2.463^ ns^	0.258^ ns^

Number of sequences (N), significant (*) at *p* <0.10, non-significant (ns)

The expected heterozygosity (*H*_*e*_) ranged from 0.120 (Boran**-**Holstein) to 0.256 (Boran). Boran**-**Holstein cattle had the lowest number of PIC (0.101), whereas in Begait the highest numbers of PIC (0.2005) was detected. MAF values ranged from 0.078 in Boran**-**Holstein to 0.206 in Begait cattle. The different sequences of *DGAT1* gene were observed in heterozygote excess in studied breeds that ultimately lead to low and negative *F*_*IS*_ values (Table 2). Fu’s *Fs* was negative and statistically non-significant (*p* > 0.10) in Fogera and Horro cattle breeds, while positive in Boran and Boran**-**Holstein. The Tajima’s *D* value obtained was negative and statistically non-significant (*p* > 0.10) in Boran, Fogera, and Horro, while positive in Boran**-**Holstein. Both tests are positive and statistically significant in Begait cattle (Table 2).

### *DGAT1* gene population differentiation among Ethiopian cattle breeds

Two main parameters (*F*_*ST*_ index and exact *G* test) for the inspection of population genetic structure and differentiation levels among cattle breed were used. The *G*_*ST*_ (coefficient of interpopulational genetic differentiation) test which is used to measure among-population differentiation, relative to the total diversity value, was compared between cattle breeds and highest value was observed between Boran and Begait (0.096). The lowest *G*_*ST*_ value (**-**0.016) was found between Boran and Fogera (Table 3). The pairwise *F*_*ST*_ showed significant differences across all cattle breeds (*F*_*ST*_ = 0.13; *p-*value ≤ 0.0001).

**Table 3:** Genetic distances between pairs of populations based on Wright’s F-statistics *F*_*ST*_ below the diagonal and Nei’s genetic distance *G*_*ST*_ above the diagonal estimated

	BHC	BR	BG	FG	HR
BHC	0	0.03659	0.07786	0.06447	0.05644
BR	0.06655	0	0.09618	-0.01613	-0.02029
BG	0.34631	0.21648	0	0.07531	0.08628
FG	0.15855	-0.02984	0.13728	0	-0.02013
HR	0.12778	-0.03543	0.25748	-0.01595	0

**Fig. 2:**
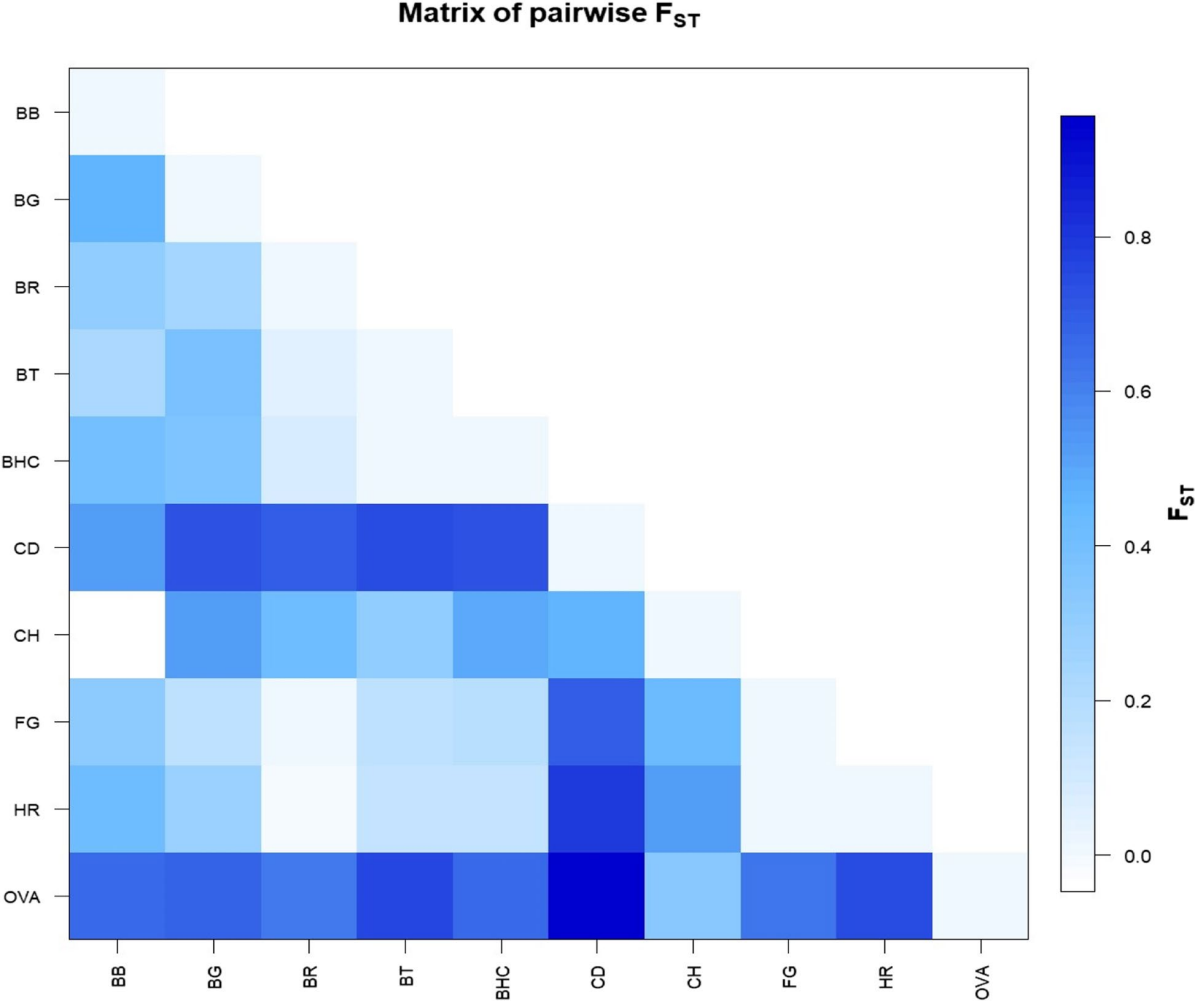
DGAT1 gene graphic representation of calculated FST values between pairs of population (Bos taurus (BT), Camelus dromaderies (CD), Capra hircus (CH), Ovis aries (OVA), and Bubalus bubalis (BB ), generated by the R function: pairwise FST matrix.R

The estimated *F*_*ST*_ values between *Bos taurus* breeds and individual cattle breed considered in this study ranged from 0.005 (*Bos taurus* vs. Boran**-**Holstein) to 0.389 (*Bos taurus* vs. Begait). Among the Ethiopian breeds, the *F*_*ST*_ value was relatively high (*F*_*ST*_ = 0.346) between Begait and Boran**-**Holstein, and a low *F*_*ST*_ value of **-**0.029 was noted between Boran and Fogera. Moreover, *F*_*ST*_ values were computed across species leading to the detection of higher genetic differences and *Bubalus bubalis* and *Camelus dromaderies* showed least differentiation and maximum differentiation was observed between cattle and other species (Fig. 2).

### Phylogenetic relationships and Median Joining network

The evolutionary history was inferred by using neighbor joining method based on the Tamura**-**Nei model with bootstrap replications of 1000 and the evolutionary tree inferred from cattle.

**Fig. 3:**
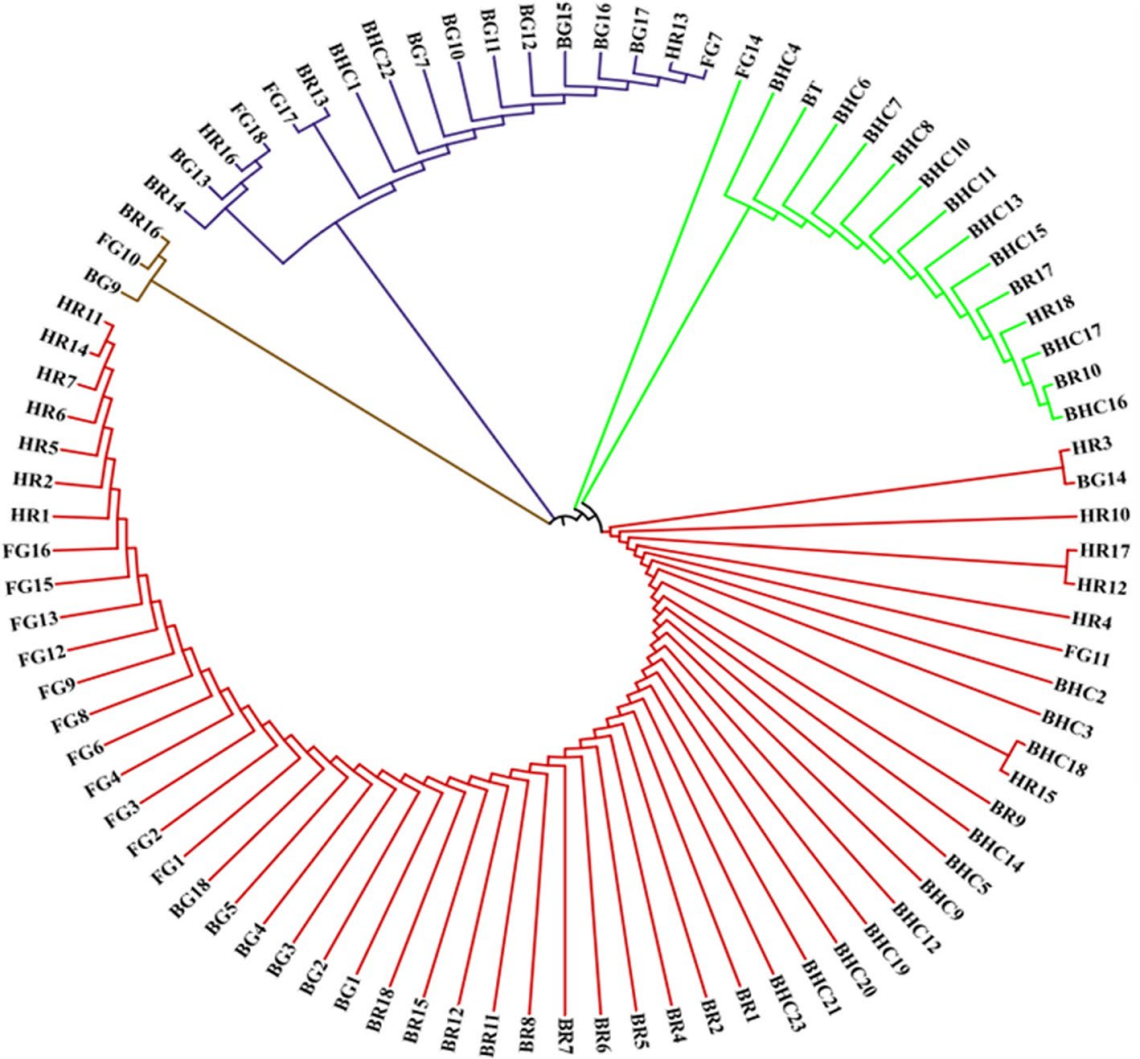
Neighbor-joining tree showing the genetic relationships among 89 DGAT1 gene sequences grouped into four distinct clusters using evolutionary distances computed by the Nei (1993) method. The labels were coded in such a way that the first two/three letters stands for breed name and the number is order of the different breeds. The four colours represent the four clusters

**Fig. 4:**
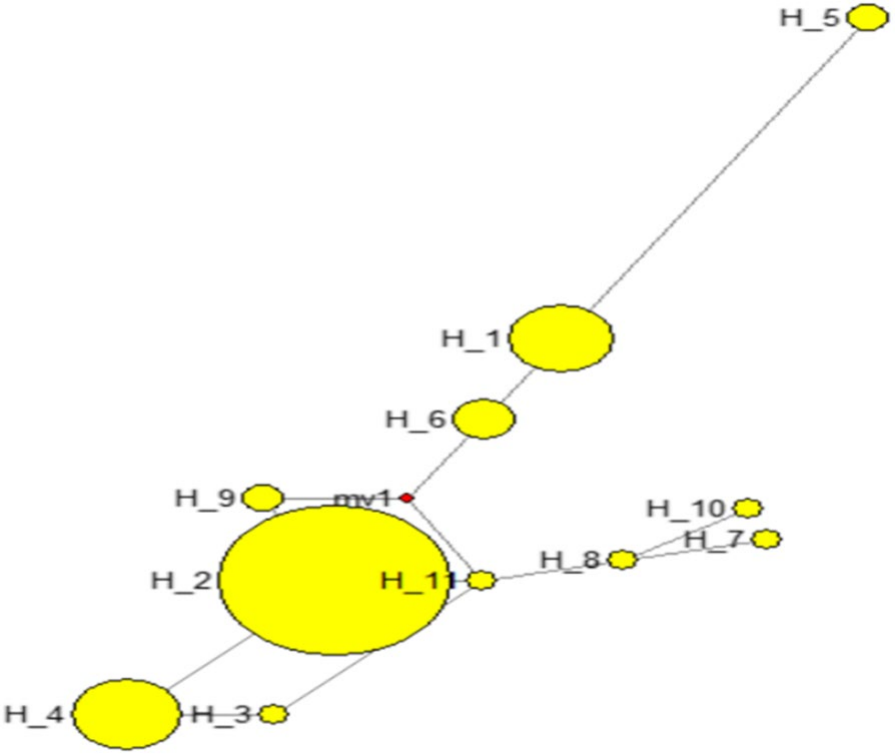
*DGAT1* Median-joining network constructed by NETWORK software version 10.0.0, and yellow circles represent the number of sequences that have sizes proportional to the frequencies. The branch length is proportional to the mutation rate of the haplotypes whereas the red diamond represents the median vector

*DGAT1* locus sequences are presented in Figs. 3 and 4.

The analysis revealed four separate clusters among the studied cattle breeds sharing a common node from the constructed tree (Fig. 3). It looks that *DGAT1* alleles might have evolved through multiple lineages.

Furthermore, a median-joining network tree was constructed based on the haplotypes (Fig. 4). The number of individual sequences in H-1, H-2, H-3, H-4, H-5, H-6, H-7, H-8, H-9, H-10 and H-11 was 11,53,1,12,2,4,1,1,2,1,and 1, respectively. Here, H11 being with most probable ancestral haplotype. Haplotypes H5, H7 and H10 separated from the rest of the clusters.

## Discussion

The reported haplotypes number, haplotype diversity and nucleotide diversity were 11, 0.615 and 0.0100 respectively. These values are significantly higher than the values reported by Faraj and his colleagues [8] where number of haplotypes, haplotype diversity and nucleotide diversity of *bos indicus* cattle were 2, 0.536 and 0.0031, respectively. The combination of high haplotype diversity and low nucleotide diversity, suggested small differences between haplotypes and can be a signature of a rapid population expansion from a small effective population size [22]. The unique breeding histories of some population may account for the high variability of genetic diversity. The ratio of (Ka/Ks) was evaluated and the values recorded were less than one for all the breeds. We found that cattle *DGAT1* gene have been subjected to purifying selection (Ka/Ks = 0.00–0.708). The average Ka/Ks ratio for *DGAT1* gene was 0.564, indicating that the evolution of bovine *DGAT1* is largely shaped by strong purifying selection through the removal of alleles that are deleterious, resulting in stabilizing selection in the phenotypic outcomes. It has been shown that a Ka/Ks ratio of or close to 1 indicates no strong selection pressure, a ratio larger than 1 indicates that the protein is subjected to positive selection, whereas less than 1 indicates the presence of purifying selection [23]. Positive selection is observed less often than purifying selection and most mammalian genes are under strong to moderate purifying selection [23]. *DGAT1* genes were under purifying selection in Ethiopian cattle. Purifying selection against newly arising deleterious mutations is essential to preserve biological function of *DGAT1* gene among Ethiopian cattle.

Minor allele frequency(MAF) refers to the frequency of the second most common allele in a population, and it affects heritability and predictive ability and multiple studies have shown that MAF affects predictive ability [24]. Higher MAF recorded for indigenous cattle than Boran-Holstein. The higher values for indigenous breeds can be explained by the fact that loci used in this study were detected in indicine breeds, and their average MAF was much lower in taurine**-**indicine breeds. The present MAF values were lower than the report of Edea and his colleagues [25] for Ethiopian cattle. This could be attributed to the differences in genotyping platforms used and causal variants have lower MAF than SNPs in a panel [26].

The heterozygosity situation in the present study for *H*_*e*_ ranged from 0.120- 0.256 were in the previously reported range of 0.02 to 0.50 for *Bos indicus* cattle [9]. Observed heterozygosity at *DGAT1* locus in this study was higher than the reports of Borgou (0.388) and White Fulani (0.155) cattle breeds of Benin [11]. The values of heterozygosity were lower when compared with previous study on Rathi, Sahiwal and Kankrej cattle breeds of India [27]. Similarly higher values of heterozygosity in Holstein cattle breeds was reported by previous studies (*H*_*o*_ ranged from 0.313–0.938 and *H*_*e*_ ranged from 0.264–0.498) [28]. The highest observed heterozygosity and haplotype diversity in Begait indicates that Begait cattle are more genetically variable at the *DGAT1* locus compared to the other breeds.

A marker with PIC > 0.5 can be considered as highly informative, whereas, 0.5 > PIC > 0.25 recognized as reasonably informative and below 0.25 is measured as slightly informative [29]. In the present study breeds PIC values range between 0.101 and 0.200. Thus, the marker is slightly informative for the studied breeds.

Genetic divergence among populations of the same or different breeds is usually quantified by fixation indices or F statistics [30]. The *F*_*IS*_ coefficients are the classical Wright’s F-statistic, which estimates the variation within populations. Specifically, it measures the reduction in heterozygosity in an individual caused by nonrandom mating within its subpopulation [30]. The *F*_*IS*_ coefficient value was negative for the studied breeds ranged from **-**0.284 to **-**0.606. The present study is in agreement with the findings of Rincon et al*.* [10] who also observed negative estimates of *F*_*IS*_ for *DGAT1* loci in Uruguayan Creole cattle population and for Borgou cattle of Benin [11]. Negative estimates of *F*_*IS*_ coefficient value for Ethiopian cattle were also observed [25]. This suggests there is no heterozygosity deficiency in all studied cattle population as a result of uncontrolled mating leading to higher diversity.

The negative *F*_*ST*_ values recorded by Fogera and Horro with Boran as well as between Horro and Fogera indicated that genetic subdivision could not be established among these populations. Moreover, the genetic differentiation of all the breeds based on *G*_*ST*_ was small and supported by the previous studies [25, 27]. The little genetic distance inferred from the breeds could be the result of the evolutionary or domestication history of cattle breeds [31] and which could be due to their common ancestral origin.

Neutrality tests of Tajima’s *D* [32] and Fu’s *Fs* statistics [33] were carried out to assess signatures of recent historical demographic events. Tajima’s *D* test is based on comparison of the allelic frequency of segregating nucleotide sites, while Fu’s *Fs* test is based on the alleles or haplotypes distribution [32, 33]. They estimate the deviation from neutrality, which is based on the expectation of a constant population size at mutation-drift equilibrium and negative values of both tests signifies an evidence for an excess number of alleles and could be expected from a recent population expansion or genetic hitchhiking and positive value signifies deficiency of alleles, as would be expected from a recent population bottleneck and/or balancing selection [32–34]. Overall Tajima’s *D***(-**0.147) and Fu’s *Fs***(-**1.534) tests statistics in all populations were negative and statistically non-significant(*p* > 0.10) and consistent with a populations in genetic equilibrium or in expansion [35]. However, the two tests of neutrality were not statistically significant for Boran**-**Holstein, Boran, Horro and Fogera cattle populations (Table 2). The results suggested that these populations are in genetic equilibrium or in expansion and Tajima’s and Fu’s neutrality tests were both significant for Begait population, indicate deficiency of alleles, as would be expected from a recent population bottleneck (Table 2). The Begait cattle have been reduced in population size; however, they maintained genetic diversity which is comparable to other studied breeds. The influence of factors which affect genetic diversity can complicate an attempt to interpret the genetic diversity of any population in terms of population size as observed in Begat cattle populations [36]. We observed a close genetic relationship between the Ethiopian cattle breeds from the inferred phylogenetic tree and median joining-network tree. The current result is consistent with the report of other researchers [25, 37]. This is generally interpreted as indicative of a population that has recently expanded in size from a small number of founders following a population bottleneck [31].

## Conclusions

The overall diversity indices showed the cattle populations studied were polymorphic for D*GAT1* locus. *DGAT1* genes were under purifying selection and the presence of high gene diversity, heterozygosity and polymorphic information content revealed sufficient genetic variation in the studied cattle breeds. Fixation indices indicated significant differentiation among all the breeds. This study confirmed that the *DGAT1* gene locus is extremely crucial and may provide baseline information for in-depth understanding, exploitation of milk linked gene variation and could be used as a marker in selection programmes to enhance the production potential and to accelerate the rate of genetic gain in Ethiopian cattle populations exposed to different agro ecology condition.

## Methods

### Sampled populations and genomic DNA extraction

A total of eighty nine animals comprising of five Ethiopian cattle (Boran, *n* = 17; Begait, *n* = 16; Fogera, *n* = 17; Horro, *n* = 16 and Boran**-**Holstein, *n* = 23) were considered in this study (Table 1). Animals were selected randomly from each breed with intentional exclusion of closely related ones. Blood samples were collected from the tail head with a volume of 4 ml under aseptic conditions and gently mixed with ethylene diamine tetraacetic acid (EDTA) anticoagulant placed into an ice box containing ice. Extraction of genomic DNA was carried out using modified salting out extraction procedure [38]. Briefly, blood samples were first thawed at room temperature and about 500 µl blood was poured into 2 ml centrifuge tube. Exactly 800 µl of lysis buffer (0.3 M sucrose, 0.01 M Tris Hcl, pH7.5, 5mMmgcl and1%tritonX100) was added into the centrifuging tube and blood samples were mixed gently by inversion. Then it was centrifuged for 5 min at 4725 rpm. The supernatant was removed carefully and the step was repeated until white pellet obtained. The pellet was vigorously vortexed and re-suspended with the 60 µl of 10 mM trisHcl pH 8 and centrifuged again at 2500 rpm for 2 min. After discarding the supernatant, the pellet was re-suspended in 66 µl 10 mM tris Hcl, 66 µl laundry powder solution (30 mg/ml laundry powder solution) and glass beads and vortexed for 2 min. Exactly 50 µl of 6 M Nacl was added and vortexed again for 20 s, then centrifuged for 5 min at 11573 rpm and the supernatant was transferred into 2 ml eppendorf tube and 150 µl of 96% ethanol was added and the solution was mixed by inversion and centrifuged for 3 min at 13,000 rpm until the genomic DNA was precipitated. The precipitate was then rinsed twice with 100 µl of 70% ethanol in 1.5 ml eppendorf tube by inverting the solution for 5 min and centrifuged for 2 min at 12,000 rpm. Then ethanol was carefully poured off using tissue paper. The DNA was allowed to air dry for 30 min. Finally, the extracted DNA was allowed to dissolve in 60 µl TE buffer (10 mM tris–Hcl pH 8; 0.1 mM EDTA, pH 7.4) overnight and stored at 4^0^C. The quality of the DNA and its concentration were quantified via NanoDrop1000 and electrophoresis in 1.7% agarose gels. Those DNA samples with good quality and quantity were considered for amplification and sequencing.

### PCR Amplification and Sequencing of *DGAT1* Gene region

To amplify 278 bp product size of *DGAT1* exon 8 region, primers were designed with accession number AJ318490 as reference sequence using primer 3 plus software [39]. The region was amplified by PCR using the two primers: Forward 5’-AAGGCCAAGGCTGGTGAG -3' and Reverse: 5’-GGCGAAGAGGAAGTAGTAG -3'.

Polymerase chain reaction (PCR) was carried out in a total volume of 25 μL containing, 5X PCR buffer (5 μl), 1.5 mM MgCl2 (3 μl), 10 Mm dNTP’s mix(1 μl), forward primer 70 pmol/μl (0.5 μl), reverse primer 70 pmol/μl (0.5 μl), genomic DNA 25 ng/ μl (2 μl), Taq DNA polymerase 5U/μl (0.3 μl) and DNAase free water (12.7 μl). The optimized thermal profile include an initial denaturation at 94 °C for 3 min, 30cycles of denaturation at 94 °C for 1 min, annealing at 57 °C for 45 s, elongation at 72 °C for 1 min and a final extension at 72 °C for 7 min. Finally, the PCR products were visualized post electrophoresis on 1.7% agarose gel with acetate EDTA (TAE) buffer followed by GelRed staining. For sequencing, the PCR products were sent to Konkuk University, Seoul, South Korea.

Before sequencing, sequencing reaction was performed for the PCR products by using one of a pair of PCR primers used for amplification of the *DGAT1* (Forward) gene as the size was short (278 bp). After completion of the reaction, reaction products were purified using a sodium acetate–ethanol purification method. The purified products of sequencing reactions were analyzed on an ABI3730 capillary genetic analyzer (Sanger Sequencing Machine). Finally, the sequences were analyzed and deposited to GenBank with the following accession numbers (ON262825**-** ON262849).

### Data management and statistical analysis

Genetic diversity at sequence level was performed encompassing partial intron 7, exon 8 and intron 8 region of *DGAT1* gene. Prior to analysis, all the chromatograms were visualized and sequence fragments were edited using Bio-edit version 7.0.5.3 and aligned by clustalX2 software package [40]. DNA polymorphism, observed and unbiased expected heterozygosity was computed using ARLEQUIN software version 3.5.2.2 [41]. Minor allele frequency (MAF), Polymorphic information content (PIC) and coefficient of inbreeding (*F*_*IS*_) was calculated using Power Marker (version 3.25) [42]. Evolutionary analysis of *DGAT1* exon 8 regions was carried out through analysis of rates of synonymous (Ks) and non-synonymous (Ka) substitutions. Ka/Ks ratio, the average rates of non-synonymous (Ka) over synonymous substitutions (Ks) per site were computed using DnaSP v5 [43].

Population differentiation due to the population genetic structure was also assessed from sequence data, population pairwise Wright’s *F*_*ST*_ [44] values were calculated by using ARLEQUIN software version 3.5.2.2 applying 1000 replication values [41]. The pairwise *F*_*ST*_ graph was displayed by Rcmd (console version of the R statistical package) installed on the computer integrated with ARLEQUIN. Moreover, to strengthen the analysis fifteen haplotypes/sequences of milk producing farm animals from Genbank including *Bos taurus* (AJ318490, EU077528, MF069174 and MF445056), *Bubalus bubalis* (MZ230553, MZ230553, MF069172 and KX965992), *Camelus dromedaries* (MF069170 and MF069171), *Capra hircus* (LT221856 and FJ415876) and *Ovies aries* (KJ918741, FJ415875 and EU178818) from Germany, Turkey, India, Iran and Benin were included in the analysis. Hence, population genetic differentiations based on the *DGAT1* genes of the breeds were evaluated by Ne’s genetic distance (*G*_*ST*_) by DnaSP software [43].

To test for past population expansion, we used two statistical tests Tajima’s *D* [32] and Fu’s *Fs* [33]. The analyses were implemented in the program ARLEQUIN software version 3.5.2.2 [41] and p-values were generated using 1,000 simulations under a model of infinite site neutrality.

Phylogenetic analysis of coding region was carried out for *DGAT1* gene in accordance to neighbor-joining algorithm [45] based on the Tamura-Nei model [46] using MEGA 11 [47] via implementing1000 bootstrap values [48]. Positions from DNA sequences containing gaps or missing data including identical sequences were excluded from the analysis. A median-joining network (MJN) tree was constructed using the NETWORK software (version 10.0.0) [49]. To evaluate the median network, the nucleotide sequences were first converted into binary data, while identical sites were omitted from the analysis. Each split was programmed as a binary character, satisfying the values of 0 and 1. The haplotypes were denoted as a binary vector in this method [49].

## Data Availability

The datasets generated and/or analysed during the current study are available in the NCBI- GenBank® repository with accession numbers (ON262825-ON262849).

## Abbreviations

*DGAT1*: *Diacylglycerol O-Acyltransferase 1*
MAF: Minor allele frequency
NCBI: National Center for Biotechnology information
PIC: Polymorphic information content
rpm: Revolution per minute
TAGs: Triacylglycerols
